# Class-Incremental Learning on Video-Based Action Recognition by Distillation of Various Knowledge

**DOI:** 10.1155/2022/4879942

**Published:** 2022-03-24

**Authors:** Vali Ollah Maraghi, Karim Faez

**Affiliations:** Department of Electrical Engineering, Amirkabir University of Technology (Tehran Polytechnic), Tehran, Iran

## Abstract

Recognition of activities in the video is an important field in computer vision. Many successful works have been done on activity recognition and they achieved acceptable results in recent years. However, their training is completely static, meaning that all classes are taught to the system in one training step. The system is only able to recognize the equivalent classes. The main disadvantage of this type of training is that if new classes need to be taught to the system, the system must be retrained from scratch and all classes retaught to the system. This specification has many challenges, such as storing and retaining data and respending training costs. We propose an approach for training the action recognition system in video data which can teach new classes to the system without the need for previous data. We will provide an incremental learning algorithm for class recognition tasks in video data. Two different approaches are combined to prevent catastrophic forgetting in the proposed algorithm. In the proposed incremental learning algorithm, two approaches are introduced and used to maintain network information in combination. These two approaches are network sharing and network knowledge distillation. We introduce a neural network architecture for action recognition to understand and represent the video data. We propose the distillation of network knowledge at the classification and feature level, which can be divided into spatial and temporal parts at the feature level. We also suggest initializing new classifiers using previous classifiers. The proposed algorithm is evaluated on the USCF101, HMDB51, and Kinetics-400 datasets. We will consider various factors such as the amount of distillation knowledge, the number of new classes and the incremental learnings stages, and their impact on the final recognition system. Finally, we will show that the proposed algorithm can teach new classes to the recognition system without forgetting the previous classes and does not need the previous data or exemplar data.

## 1. Introduction

Recognition of human activity in the video has many applications, such as human interaction with computers, surveillance systems, and monitoring of human behavior in industrial environments. Approaches based on deep learning have reported excellent results in recognizing activity, especially human activity. But using these methods in real and dynamic environments such as urban space, wildlife, and industrial environments faces challenges. Because it is always possible to see new activities that the existing recognition system has not already seen, in this case, there is a need to update the system constantly.

Deep learning has significant achievements in terms of recognition and classification [1–3], but it also has some constraints. Training a model with a deep learning approach requires many annotated data and computational resources due to its increasing depth and complexity. Most successful methods restrict the batch setting: data are provided before training; hence, optimizing metaparameters and model selection is usually based on a complete dataset. Also, the training can rely on the assumption that the data and its underlying structure are static. But this is contrary to real-world scenarios. In the actual situation, training data is not available for all classes initially, and the number and nature of classes are not necessarily clear. A classification system in real environments should learn new classes incrementally when their training data becomes available and, of course, does not undermine the recognition of previous ones. But the main problem is that training a neural structure using only new classes' data leads to forgetting previous classes, called catastrophic forgetting.

On the other hand, training a system from scratch to learn new classes faces challenges, including the high cost of training, storing previous class data for future training, and perhaps privacy restrictions for maintaining some data. So, as computer vision moves closer towards artificial intelligence, more flexible approaches are needed to handle the large-scale and dynamic situation more felt. The purpose of this work is to learn new categories to model without the need for previous data and without reducing the model's performance about previous classes, known as class-incremental learning in literature.

This paper provides a class-incremental learning method for recognizing human action in video data. The base model is an RNN-based deep neural structure, given the achievements of deep learning in classification, that should learn human action classes in video data. Class-incremental learning for RNN-based deep structures has rarely been done. The proposed method combines two common approaches in incremental learning (network sharing and network knowledge distillation). Using the proposed algorithm, we will show that new classes can be taught to a human action recognition system without data from previous classes. Of course, the system's overall performance will not be greatly impaired. We propose the distillation of network knowledge in two levels of classification and feature. The feature section can be decomposed into two sections of distillation of temporal and spatial features. We also proposed using Temporal Segment LSTM (TS-LSTM) for the temporal representation of the video instead of the usual use of LSTM. This technique helps to better temporally represent the video and improve the recognition system's performance. The proposed method will give comparable results to exemplar-based methods without the use of exemplar data. Meanwhile, in the Related Works section, we show that the class-incremental learning of action recognition in video is rarely done without the exemplars. We evaluate the proposed algorithm and compare it with other methods based on exemplar data, and we show that the proposed method has performed better. Under similar conditions, the recognition accuracy has improved by almost 8% and 6.5% compared to the existing methods on the UCF101 and HMDB51 datasets.

The innovation of this work is in three cases: first, network distillation at classification and feature levels, where distillation at the feature level is done separately for spatial and temporal features, second, providing class-incremental learning in video data without storing data or exemplars, and third, initializing new classifiers using previous classifiers to learn new classes faster and better. Also, our base model is an RNN-based structure, and other existing methods are not effective for these structures.

In the rest of the paper, we review some related works in Section 2. The proposed algorithm is presented in Section 3. We present the experimental results and evaluations in Section 4, discuss results in Section 5, and conclude the paper in Section 6.

## 2. Related Works

### 2.1. Incremental Learning on Video Data

Class-incremental learning means that a classifier architecture can accommodate new classes at any time during the training process without requiring access to all training data seen so far. The minor works in incremental learning in video data are almost related to domain-incremental learning [4]. Class-incremental learning to recognize human action in video data is suggested using a recursive-tree structure [5], which adds one class to the tree as each new class's data is added.

In deep learning, very little work has been done on class-incremental learning in video data, especially on action recognition in video. The adaptive RNN tree is proposed for large-scale action recognition, adapted to new classes by augmenting an existing model [5]. The ODN (Open Deep Network) [6] detects new categories by applying a multiclass triplet thresholding method and dynamically reconstructs the classification layer. It opens the deep network by continually adding predictors for new classes. The weights of the new classifiers are initialized from the average weights of the previous classifiers. The new model is fine-tuned using both new and old samples. The goal is to reduce the training energy from scratch and still require previous data. A temporally attentive knowledge distillation method is presented, which distills the network knowledge over the network and intermediate layers and uses it to prevent catastrophic forgetting [7]. Importance weights are estimated during training, and knowledge is distilled from these importance weights during incremental learning. This method also uses the storage of exemplar data for use in incremental learning. To improve the performance of incremental learning based on network distillation, the spatial-temporal feature decomposition into two parts, spatial and temporal features, is presented [8]. A dual granularity exemplar selection method is proposed to select exemplar data from previous classes for use in incremental learning [8].

As mentioned, little work has been done on incremental learning in video data. The exemplar data storage of previous classes is also usually used. On the other hand, the deep structures used for classification tasks in a single image are similar to video classification's deep systems. Therefore, catastrophic forgetting is a common phenomenon in the image and video domains. So, reviewing incremental learning in single images can give us a better insight into this issue.

### 2.2. Incremental Learning on Image Data

Mensink et al. [9] showed that the nearest class mean (NCM) classifier could do so. NCM represents each class by the average feature vector of all examples observed for the class so far. NCM has performed well for incremental learning and is more robust than other standard parametric classifiers [9–11]. The prototype-based classification idea of NCM was adapted for incremental classifier and representation learning (iCaRL) [12] where the average vector is computed only over a specifically chosen subset of all examples. The dilemma of prototype-based methods deals with complex classification problems, where the number of classes increases and their nature becomes more complicated.

The recent success of deep neural networks can be mainly attributed to their suitable data representation [13–16]. The main problem in learning representation in the incremental learning mode is catastrophic forgetting, introduced by McCloskey and Cohen in 1989 [17]. Training a neural network with new data causes the previous data to be forgotten. Inspired by the idea of transfer learning [18], the previous model's knowledge can assist in learning new related classes. It has been shown that the features extracted in the lower layers of CNNs are general, which can be used in various domains, similar to Gabor filters and edge or corner detectors [19]. In contrast, the extracted features from the end layers are more abstract and specific for a class. This feature of CNNs is the basis of the transfer learning and partial sharing approach for incremental learning [20].

Much of what has been done recently for incremental learning in neural networks is based on the freeze/growth scenario [20–22]. Splitting the base network into various subnetworks and creating a tree structure are proposed to incrementally learn the new classes [23, 24]. An adaptive hierarchical network composed of DCNNs is proposed for incremental learning that grows and learns as new data becomes available [25]. In [26], the Bayesian transfer learning algorithm is proposed to avoid retraining the whole model from scratch. Selecting a portion of the initial exemplar data and storing it for training new classes are also provided to prevent catastrophic forgetfulness [12, 27, 28]. This scenario requires additional resources to store exemplar data. Using the generative adversarial networks (GANs) to create fake data is proposed [29, 30]. This approach eliminates the need for additional resources to store data but leads to additional training GANs. A method based on bias correction is proposed to solve the educational data unbalancing issue [31].

Network knowledge distillation has been used to reconstruct previous classes' features and train new classes to solve the catastrophic forgetting problem [32, 33]. In [32], the input is given to the previous model to extract the previous classes' information in the input image. The model responses to inputs are used as the ground truth for old classes during training the new classes to the system [14]. Castro et al. [34] proposed that, to train the new class, use network knowledge distillation to calculate the output of prior categories and generate a set of logits. These logits are then used to calculate the loss in training new classes to force the network to retain information from previous classes. An attentive feature distillation approach was proposed to distill necessary knowledge using both top-down and bottom-up attention for incremental learning object detection [35]. Incremental learning in the semantic segmentation of objects is also proposed with the same approach [36]. This approach is also used for incrementally object detection tasks [37] to train the class of new objects.

A review of related work suggests that if the main goal is to classify with one output class for each input, the appropriate approach is network sharing or storing and reproducing some of the data from previous classes [12, 20–24, 27–30]. For tasks with multiple outputs per input, such as multiple object detection, the use of network knowledge distillation may be a good approach, given the possibility of having information about multiple classes in a single input [35–37].

## 3. Proposed Approach

The purpose of this work is class-incremental learning for action recognition in video. A review of related work showed that the appropriate approach for class-incremental learning in single-output tasks is network sharing or storing and reproducing some of the data from previous classes [12, 20–24, 27–30]. The network distillation approach is more suitable for tasks where the input includes multiple classes, such as multiple object detection and multiclass segmentation. The new class data may consist of parts of some previous classes in these cases. So, the distillation network distills the information about those classes, which helps preserve previous network information in new training. However, these conclusions are for single-image data and may not be entirely valid for video data.

Our work is to recognize the action in the video, which is a single-class classification task. A specific action may consist of several subactions, some of which may be present in other activities. The way action recognition algorithms work in video is usually that first low- and high-level features are extracted from video frames (usually by a CNN). Recursive structures represent the temporal dependence of these features, and, finally, the classification is based on this representation. The extracted features in the first step are abstract visual features, and, due to a large number of frames in the inputs, the feature extraction module becomes general. In other words, the feature extraction module obtained from the training of the early classes also can extract the appropriate features from the data of the new classes. Therefore, the network sharing technique can be used for incremental learning in this work.

As mentioned, an action consists of its various components, including various subactions and abstract properties. The feature extraction module obtains abstract features in the first step. Subactions and their combinations are calculated to represent the action by recurrent structures in the second step. A new action may contain components that already exist in previous classes. Therefore, if a new action class data is applied to the model, the recurrent section may also represent different components of previous actions. In this case, the previous network information can be distilled using network distillation. Thus, although this is a one-class classification task, the network distillation approach can also be used for class-incremental learning.

### 3.1. Class-Incremental Learning

Our main goal in this work is to apply class-incremental learning to recognize the action in the video. Our proposed algorithm combines two approaches: network sharing and network distillation. After training the basic model with the data of some classes, it is assumed that the feature extraction section (CNN) can extract appropriate properties of other classes. Therefore, the feature extraction module is fixed (frozen) for subsequent training efforts. This trick implements the concept of network sharing.

Distillation of network knowledge is the extraction of information learned from the network and applying this information to the network during incremental learning to prevent forgetfulness. In fact, the previously taught model as a teacher controls training the new model (student) not to forget the previous information. The teacher model is not updated during incremental learning. Distillation of network knowledge can be applied at the classification level (probabilities generated by the classification layer) or at the feature level (feature maps obtained in the middle layers). In a simple knowledge distillation algorithm, only the output of the classification layer is used to extract network knowledge and remind it in incremental training. In this case, the student network tries to preserve past information by reproducing the values generated by the teacher model. The last layer classifiers generate a probability for each class for each video input. Before new training, these probabilities are first calculated for the new class input, and, during the new classes training, the previous classifiers are forced to reproduce the previous probabilities. Therefore, when training the student model to train the new classifiers, the previous classifiers retain previous knowledge by being forced to reproduce their corresponding values in the teacher model. The task of preventing the forgetting of past knowledge in these circumstances is the sole responsibility of the classification layer.

But the information recorded in the network is available throughout the network. By extracting this information and reminding it to the network, it is possible to help preserve the network knowledge in new learning [7, 8]. In this situation, the teacher model controls the training of the student model throughout the model, not just in the classification portion. The feature maps obtained from the middle layers in 3D neural structures for video representation form a tensor with *T* × *H* × *W* dimensions. This tensor contains spatiotemporal information obtained from video, including spatial information in dimensions *H* and *W* and temporal information in dimension *T* [8]. Separating spatial information and temporal information and distilling them has been shown to help improve incremental learning performance [8]. Pooling in temporal and spatial dimensions must be performed to decompose the tensor containing spatiotemporal information into spatial and temporal parts, respectively.

**Figure 1:**
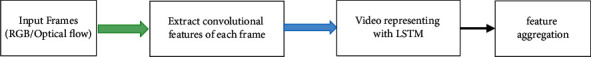
Block diagram of the process in each processing stream. At first, the convolutional features of each frame are extracted. Then, the whole input video is represented by the LSTM block. Finally, the elements are aggregated.

In our case, the model used to recognize action from the video is an RNN-based deep structure described in Section 3.2. In this type of structure, spatial features are first extracted at the frame level, and then temporal relations are represented by a recurrent part.  Figure 1 shows this process. Therefore, spatial information is available separately from temporal information at the end of the first section (convolutional feature extraction module in Figure 1). Temporal representation is also available at the output of the recurrent part (LSTM module in Figure 1). Therefore, no process is required to parse spatial and temporal information.

**Figure 2:**
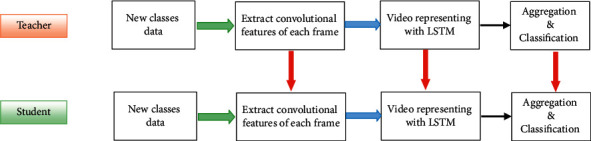
Control of student model training by teacher model at three levels of classification, temporal features, and spatial features. The teacher model is not updated during training the student model.

According to the above, the network knowledge distillation technique is used to apply incremental learning by controlling the training of the student model by the teacher model. This control is applied at three levels: classification layer, spatial features, and temporal features.  Figure 2 shows this control. Therefore, the knowledge of the network is distilled at three levels of classification, spatial characteristics, and temporal characteristics.


(4) $$\begin{array}{c} L_{cD}=\overset{F}{\underset{f=1}{\sum }}\left(-\frac{1}{N}\overset{N}{\underset{i=1}{\sum }}\overset{C}{\underset{j=1}{\sum }}{\hat{p}}_{ij}\;log\;{\hat{q}}_{ij}\right), \end{array}$$

The technique of distillation network knowledge and applying it during incremental learning is done by calculating the loss function and backpropagating it. The base network is trained using the common cross-entropy classification loss function (*L*_*cl*_). The loss function in the incremental learning phase (*L*_*inc*_) consists of two parts, one part related to classification and the second part associated with knowledge distillation. As for the base model training, the first part is a cross-entropy loss function (*L*_*cl*_) and the second part is a distillation loss function (*LD*).

(1) $$\begin{array}{c} L_{inc}=L_{cl}+\;\gamma LD, \end{array}$$

where *γ* is hyperparameter. The cross-entropy loss (*L*_*cl*_) is as follows:

(2) $$\begin{array}{c} L_{cl}=-\frac{1}{Ns}\sum_{i=1}^{Ns}\sum_{j=1}^{C}p_{ij}\;log\;q_{ij}, \end{array}$$

where *p*_*i*_ is the grand truth for sample *i* and *q*_*i*_ is a softmax score of the logits of a classifier for sample *i*. *Ns* and *C* denote the numbers of samples and classes, respectively. The distillation loss function (*LD*) used to maintain previous classes consists of two parts, classification and feature:

(3) $$\begin{array}{c} LD=L_{cD}+\propto L_{fD}, \end{array}$$

where *L*_*cD*_ and *L*_*fD*_ are the classification distillation loss and the feature distillation loss, respectively. The classification distillation loss is as follows:where ${\hat{p}}_{i}$ and $\;{\hat{q}}_{i}$ are the modified versions of *p*_*i*_ and *q*_*i*_, which are obtained by raising *p*_*i*_ and *q*_*i*_ to the exponent 1/*T*, as described in [38], where *T* is the distillation parameter. Distillation loss is calculated for all *F* previous classes. Choosing *T* > 1 forces the network to learn a more fine-grained separation between classes. This parameter is set to 2 for all experiments. Feature distillation loss can be calculated as fused spatiotemporal or separately in two parts: spatial and temporal. Due to the superiority of separate spatial and temporal distillation and separate access to these two features, we use separate distillation. So the feature distillation loss is formulated as follows:

(5) $$\begin{array}{c} L_{fD}=L_{tfD}+\beta L_{sfD}, \end{array}$$

where *L*_*sfD*_ and *L*_*tfD*_ are the spatial and the temporal terms of the feature distillation loss. These losses are calculated as follows: the Euclidean distance between the features taken from the base model and the model under training.

(6) $$\begin{array}{c} L_{fD}=\sum_{i=1}^{F}f_{ib}-f_{in}{}^{2}, \end{array}$$

where *f*_*ib*_ and *f*_*in*_ are the features obtained from the base model and new model, respectively.

So, the overall loss function formed as follows:

(7) $$\begin{array}{c} L_{inc}=L_{cl}+\;\gamma \left(L_{cD}+\propto \left(L_{tfD}+\beta L_{sfD}\right)\right). \end{array}$$

Therefore, the proposed class-incremental learning algorithm is as follows:

- A human activity recognition system is taught from scratch to recognize the initial classes. This trained model is used as the teacher model for the next training.
- The new model (student model) is created by sharing the base model and adding classifiers for new classes.
- The feature extraction module of the base model, except two last layers, freezes and is not updated during incremental learning. This work is derived from network sharing approaches.
- The student model is taught using new class data under the supervisor of the teacher model. During this training, knowledge distillation is done by supervising the teacher model by calculating the loss function according to (7).
- The new taught model will be used as the teacher model for future training. In other words, the subsequent incremental learnings are done from step (ii) onwards.

We introduce an excellent neural structure for action recognition in video data in the following.

### 3.2. Action Recognition System

An important feature of video data is temporal information in the video that can efficiently represent the video and identify the target class. But what is the best way to exploit time information? Many previous methods, which follow a two-stream ConvNets [39], use optical flow images to take advantage of temporal information in one of the processing streams [40–44]. In these methods, visual appearance in spatial and temporal flows and their relationship are not considered, which is one of the essential components in activity recognition tasks. Therefore, they will not perform well for these tasks.

Therefore, we can consider a three-stream structure that the first and second streams process on RGB images, and the third stream includes the processing of optical flow images. This three-stream structure was previously introduced for action recognition in the video [45] and modified for verb recognition through zero-shot learning on human-object interaction (HOI) recognition [46]. We use this structure as a base model to investigate the proposed class-incremental learning algorithm. The operation of this structure and each of its three streams is described in full detail in [45]. We briefly introduce it below. Each stream has three main processing steps shown in Figure 1. The processing flows of the three streams are almost similar.

 
*Patch-Based Representation.* Activity-related areas are first estimated in each RGB frame in this processing path. These action patches are then used to extract the convolutional properties of each frame. The extracted feature map is given to the LSTM block to represent the temporal relationship. The resulting output vectors are aggregated to form the final representation vector for estimating the action class.
 
*Focal Representation*. In this path, the activity-related area is first estimated (like as first path), the other regions are blurred, and a new RGB image is obtained. The convolutional properties are extracted from each frame's resulting image, and the process's continuation is the same as the first stream.
 
*Motion Representation*. Short-term information in a video clip can be represented by optical flow. The RNN block can obtain long-term information. Therefore, the third path can represent motion with optical flow. The convolution properties of each frame are extracted from the optical frame by a CNN network. The next steps are exactly like the other two streams.

The long short-term memory (LSTM) module is used as RNN to temporally represent the video. The LSTM module does the representation of the temporal relationship in the video. The extracted per frame CNN features are split into *k*-frame chunks in each input video and fed to the LSTM module. Each chunk fed to the LSTM module contains feature maps extracted from *F*/*k* consecutive frames of input video, where *F* is the total number of input frames and *k* is chunks number. Next, the LSTM outputs are fed into a densely connected feedforward neural network with a Softmax output layer, giving each chunk the probability of belonging to each C-action class. These probabilities are added for all chunks of each video to predict the class. The direct use of LSTM to consider temporal information is only similar to simple temporal pooling methods, such as mean or max pooling, due to the limited temporal dynamics of representing ConvNet-derived features [47]. It is shown that the features obtained from the frames of a video have similar representations [47]. Because a large portion of the video has the same feature representations over time, RNNs cannot learn the temporal information well. In the TS-LSTM method [47], the input sequence is divided into several temporal chunks, and their distinctive feature representations are learned. Each video is sampled into *N* frames and divided into *M* chunks. The features extracted from *F* input frames are split into *F*/*k* chunks. Temporary pooling is performed on each chunk, and then the pooled features of all chunks are fed to the LSTM module to represent the entire video. The output of this module will be used for recognition in the next dense layer. The TS-LSTM module learns the nonlinear feature combination and its segmental representation over time.

We substitute this TS-LSTM technique for the primary three-stream structure technique. The direct technique uses LSTM to represent each chunk typically, performs a recognition for each, and then sums the recognition results of all the fragments. The TS-LSTM technique, unlike the direct technique, uses all the video space for recognition, which will improve system performance. Due to the temporal pooling in each chunk, the total computations for recognizing a video are also reduced.

The results of the three introduced streams are merged to recognize the input video action class. The integration is as follows: A score is given to each class by a dense network in each stream. The total score of each class is calculated by summation of its scores in three streams. The class with the highest score is the recognized action class.

### 3.3. Initialization of new Classifiers

Creating a new model (student model) for incremental learning is done by sharing the teacher model and adding new classifiers related to new classes. The initialization of the weights of these new classifiers is usually done randomly. Thus, in the created student model, the weights of the new classifiers are without any initial training, while the previous classifiers are fully trained. To solve this imbalance, initializing the weights of the new classifiers with the previous weights can be helpful. The use of previous weighted averages to initialize new classifiers is proposed to reduce the learning time in the continuous training of categories for the open-set problems [6]. This work is not intended for class-incremental learning without the previous data and only makes learning the new model faster with more classes. Due to the nature of human action, which can consist of several subactions, using the information in the previous classifiers to initialize the new classifiers makes it easier to train and converge them.

We use this trick to initialize the weights of the new classifiers. Of course, we do not recommend the average of all the weights of the previous classifiers. It is best to use the average of the most similar classifiers for each new class. By feeding the data of each new class to the teacher model, the classifiers that produce the biggest probability for the input data are likely to be the most similar classifiers to the new class. Therefore, by estimating the most similar classifiers, the new classifier weights are initialized with the average of these weights as follows:

(8) $$\begin{array}{c} W_{N+1}=\frac{1}{M}\sum_{i=1}^{M}W_{i}, \end{array}$$

where *M* denotes the *M* similar classifiers. The weights of the new classifier are denoted by *W*_*N*+1_ and *W*_*i*_ denotes the weights of previous classifiers (from the teacher model).

## 4. Results and Experiments

We present the results of our method in this section and compare them with those of some other class-incremental learning methods and the static training mode (training all classes to the model in one stage). Our proposed algorithm does not use exemplar data storage. At the time of doing this, there was no other similar method for comparison. Therefore the performance of the proposed method was compared with those of incremental learning methods based on exemplar data storage.

### 4.1. Datasets

For human action understanding in videos, several appropriate datasets have been provided and published, such as UCF101 [48], HMDB51 [49], and Kinetics-400 [50]. We evaluated our method using the HMDB51, UCF101, and Kinetics-400 datasets.

*HMDB51* includes 51 action classes with 6766 videos collected from various sources. There are three training/test splits, like the setting in [45], and, in each evaluation stage, the mean average accuracy is reported.

UCF101 consists of 101 action classes, 13k clips, and 27 hours of video data. This dataset contains the user's uploaded videos, including camera motion and cluttered background. The videos of each category are grouped into 25 different groups based on some characteristics such as environments and camera movement. The dataset is separated into three training/test splits; in each split, 18 groups are used for training and seven groups for testing.

The third dataset is Kinetics-400, a large-scale video classification dataset containing 400 human action categories. Kinetics-400 is a YouTube-type video dataset that provides 240k and 20k 10-second videos for training and validation, respectively.

### 4.2. Implementation Details

The recognizer system has three streams: two spatial streams and one temporal stream. The two spatial CNN streams use an AlexNet architecture pretrained on UCF sports. The first stream inputs are the estimated action patches, and the proposed focal representation is fed for the second stream. For the 3rd stream as motion representation, we used the CNN network like the network architecture used by Wu et al. [31]. This motion-CNN is pretrained on the optical flow images of UCF sports. There are many options for generating optical flow images. The two most common methods are Brox [51] and TV-L1 [52], in which TV-L1 performed slightly better based on the evaluation in [47]. So, TV-L1 is used as input for the temporal stream.

The FC7 layer of three CNNs extracts a 4096-dimensional feature vector for each input video frame. After obtaining feature vectors for all F frames of the input video in three CNNs, these feature vectors are used by the RNN module for temporal representation. For the RNN module, an LSTM block with 256 hidden units is used. Two FC layers, respectively, with the number of neurons equal to 256 and the number of action classes, are used as a classifier in each stream.

For training the base model with initial classes, the learning rates are initially set to 5 × 10^−6^ for two spatial streams and 5 × 10^−3^ for temporal stream. These learning rates are divided by 10 when the accuracy is saturated. The weight decay and momentum for all ConvNets are set to 1 × 10^−4^ and 0.9, respectively. The batch sizes for all ConvNets are 8. The LSTM module is trained with an Adam optimizer and learning rate of 5 × 10^−5^.

The size of the input images is 224 × 224 for all streams. The number of sampled frames in each video is *N* = 24, and the number of temporal segments is selected as *M* = 6.

### 4.3. Experimental Results

In class-incremental learning scenario, To evaluate the proposed method, we follow the strategy used in [7]. First, the base model is trained with initial classes, and then the other classes are incrementally trained to model. For this purpose, all classes are sorted randomly, 50% classes (51 classes for UCF101 and 26 classes for HMDB51) are used to train the base model, and the remaining classes are used for incremental learning. The remaining 50 classes of the UCF dataset are taught to the model in 5 steps of 10 classes, ten steps of 5, and 25 steps of 2 in incremental learning. For the HMDB dataset, the remaining 25 classes are taught to the model in 5 stages of 5 classes and 25 stages of 1 class in incremental learning. Because the nature of the classes affects the result, these experiments are performed three times with randomly ordered classes. The model's classification accuracy is calculated on the seen classes in each step, and its average value is calculated.

The initialization of new classifiers is performed according to (8) with *M* = 5. First, the five most similar classifiers are calculated by calculating the teacher model's output on the new class's data for each new class. Their average weights are calculated and set as the initial values of the new classifier weights.

We test three scenarios for class-incremental learning based on using distillation knowledge and how much network sharing. These three scenarios are summarized in Table 1. The type of used distillation is depicted in the fourth column where cD represents distillation at the classification level and tfD and sfD represent distillation at the temporal and spatial features, respectively.

We train the model with the first available classes in experiments with the first scenario settings (3Stream-D). Then, the feature extraction module and the LSTM block are frozen, the classifiers corresponding to primary classes are discarded, classifiers for new classes are added, and the model is trained for new classes. During the training of new classes, only the new classifiers are taught. This experiment is just a network-sharing technique, and there is no distillation.

For the second scenario, as in the first scenario, the base model (teacher) is first trained for the primary classes. The student model is created by sharing the teacher model and adding the new classifiers. In this scenario, during incremental learning, the trained classifiers of the teacher model are not discarded. ConvNets is frozen in all streams. The distillation type is only a classification distillation. In other words, in (7), the alpha parameter is equal to 0.

The third scenario is similar to the second scenario and uses network knowledge distillation, except that, in this scenario, the distillation loss is calculated by classification and temporal features distillation. This increases the control of the teacher model on the student during incremental learning. In this case, spatial distillation is not used, and *β*=0∝ in (7), while ∝>0  is used.

Table 2 shows the class-incremental performance on UCF and HMDB datasets and compares the above scenarios with three other methods. The TCD [7] is proposed for video data, but UCIR [53] and PODNet are proposed for image datasets that are evaluated for video in [7]. All these three methods use exemplar data storage for incremental learning. The proposed algorithm does not use exemplar data. Still, for better comparison with the compared methods, the performance of the proposed method using exemplar data is also tested and is reported in Table 2.

**Figure 3:**
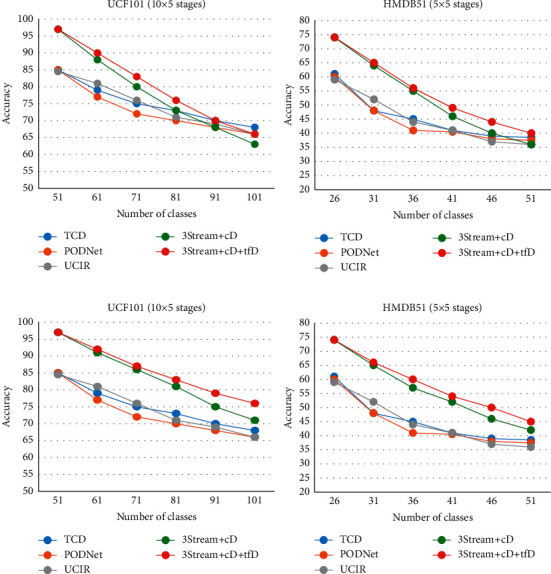
Accuracy chart in terms of incremental learning step. The top row does not use exemplar data for the proposed method, but, in the bottom row, the proposed method also uses exemplars. Compared methods use exemplar data in all cases. (a) 3Stream methods without using exemplars and (b) 3Stream methods use exemplars.

The results of Table 2 show the efficiency of the proposed algorithm. The proposed algorithm has a much higher performance than other methods if it uses exemplar data. If exemplar data are not used, the proposed algorithm still performs better or is similar to the compared methods. Of course, part of this better performance is due to the structure used, which in static learning mode is more accurate than the compared methods. In other words, the accuracy of the used model before applying incremental learning is better than the compared models at the same time. However, Figure 3 shows that the rate of accuracy drop is acceptable by applying incremental learning despite not using the previous sample data. The curves in Figure 3 show that the final model obtained by the proposed algorithm performs better than other methods if the exemplar data are used. Also, if exemplar data are not used, the performance of the final model is acceptable compared to exemplar-based methods.

For the first scenario (3Stream-D), a large part of the network (ConvNets and LSTM) is frozen. Just the parameters of classifiers are updated, so the network learning capacity is reduced. Hence, the model's accuracy on the new classes is not very high. In the final model, the extractor modules and the primary classifiers have not changed during incremental learning, and the accuracy on the primary classes has been high. Still, in the final model, the classifiers related to the primary and secondary classes have not been trained relative to each other. If the model with all classifiers is considered, the error rate increases, and therefore the overall accuracy is not very high.

For the second scenario (3Stream + cD), the base model is precisely the same as the first one. The accuracy of the final model on all classes is increased. This improvement is because network knowledge distillation has been used, and primary classifiers have been updated against new classes. Updating the LSTM block probably reduces the model accuracy on the initial classes after incremental learning. Still, due to the distillation of network knowledge, this performance reduction is not expected to be very large. Of course, it should not be forgotten that this performance improvement is in exchange for a slight increase in training energy during incremental learning. However, the energy of the error backpropagation in the recursive structures is much less than that of the convolutional structures, and, due to the freezing of the convolutional parts, the training energy is much less than that of the static training. More control of the teacher model over the training of the student model in the third scenario (3Stream + cD + tfD) causes the previous information to be less affected by the new information, and the accuracy of the model decreases less.

#### 4.3.1. Evaluation for Large Dataset

The above tests and evaluations were performed on the common UCF101 and HMDB51 datasets. Still, these two datasets do not have a large number of classes, and a larger dataset is needed to conclude situations with a large number of classes. The Kinetics-400 dataset is a large dataset that contains 400 action classes. The evaluation of the presented algorithm on this dataset can be useful for examining conditions with a large number of classes. However, since other methods have not been evaluated on this dataset and their performance has not been reported, we evaluate the results only with the proposed method and the degree of the models' accuracy with incremental learning steps.

In this case, 300 classes have been randomly selected and used to train the base (teacher) model. The remaining 100 classes are taught in incremental learning in various steps to the model. The final models of incremental learning results are also compared with the results of static training in which all classes are taught in one step. The experiments were performed three times, and the average results were reported.  Table 3 shows the evaluation results of the proposed method on this dataset.

The important columns in Table 3 are the results of the final models and their comparison with the base model and the static mode training. These results show that the use of knowledge distillation outperforms network sharing methods. Also, the knowledge distillation from different levels of the network reduces the attenuation of the model and increases the final accuracy. Reducing system performance is reasonable if the exemplar data are not used, but, given the knowledge distillation, the accuracy of the final model is not disappointing. Also, the results of Table 3 show that the number of incremental learning stages and the number of classes in each stage affect the performance of the final model. In general, the fewer incremental learning stages and the fewer new classes added to the model in fewer stages, the better the performance of the final model.

#### 4.3.2. Effect of Initialization

To initialize the new classifiers, we propose using the average weights of the most similar old classifiers. In this experiment, we evaluate the effect of this technique on system performance with equal epochs in training. We used the UCF101 dataset for this evaluation.  Table 4 shows the recognition accuracy of the final model.

The results of Table 4 show that using the average weights of the most similar classifiers to initialize the new classifiers makes the final performance of the model better than the random initialization mode in equal training epochs.

#### 4.3.3. Spatial Features Distillation

In experiments performed so far, we have not used distillation of spatial features, and we have used distillation at the level of classification and distillation of temporal features. Here we also evaluate the impact of using spatial features on incremental learning. The exemplar data are not used in incremental learning. The results of recognition accuracy are reported in Table 5.

As expected, the results of Table 5 show the positive effect of spatial feature distillation on the system's final performance. Distillation of spatial features is applied only to the first part (CNNs), and its training is separate from the rest of the network. On the other hand, the spatial part of the system includes the convolution structure, which is costly to train. Therefore, it is not unjustifiable to put it aside by accepting the slight decline in final accuracy.

## 5. Discussion

This work's primary goal is to train new classes to a recognizing system, provided that the system's performance is not impaired for the previous classes. We tested three different scenarios and compared them with some other methods. Experiments and comparisons with other methods have shown that adding distillation of network knowledge at the feature level helps increase class-incremental learning accuracy. Distillation of knowledge at the feature level at both temporal and spatial feature levels increases recognition accuracy (see Table 5). In the absence of exemplar data, our evaluations show that the proposed method can still have results comparable to exemplar-based methods (Tables 2–4).

We also investigated the effect of classifier initialization and showed that using the most similar classifiers is helpful for initialization (see Table 4).

Evaluation on large Kinetics-400 dataset showed that the fewer incremental learning stages and the fewer new classes added to the model in fewer stages, the better the performance of the final model.Although more classes in incremental learning lead to a greater decline in system accuracy, several incremental learning also increase system decline.

Experiments have shown that distillation from different model parts can help retain prior information during the training of new classes and reduce the model performance drop. Of course, this may increase the cost of training. Comparing the proposed method with other methods that use exemplar data, it has been observed that the proposed method has a similar or even better performance without using exemplar data.

## 6. Conclusion

In this research, we proposed an algorithm for class-incremental learning to an action recognition system based on deep learning that does not greatly impair the recognition accuracy of previous classes by training new classes to the system. The importance of this work is reflected in the reduction of system retraining energy for all classes and the lack of need to maintain data from previous classes for future training. In this research, a three-stream structure is used, swhere each stream includes the feature extraction section, a recurrent section, and classifiers. The main idea in this work is to maintain the network knowledge related to the previous classes and also to use this knowledge in training new classes, which helps the network not forget the previously learned information. Extracting network knowledge and reminding it to the model are done in different network parts and levels.

Our focus in this research is the distillation of network knowledge, during which the network knowledge is extracted using the training data of new classes. While learning new classes, this extracted knowledge is used to prevent the network from forgetting previous classes. Distillation of network knowledge is done at the level of classification and feature, which at the feature level can be decomposed into two parts, spatial and temporal. We also suggest that the average weights of the most similarly trained classifiers be used instead of random values to initialize new classifiers. We have shown that this low-cost work improves system performance.

Other basic structures that perform better for incremental learning for future work can be used. In this work, the classes added in the incremental learning phase are from the same dataset of classes used for initial training. Incrementally learning classes from one dataset to a system taught with another dataset is a challenge that needs to be addressed. Also, we intend to integrate this work with our previous work in the field of zero-shot learning recognition of human-object interaction (HOI) recognition [46].

## Figures and Tables

**Table 1:** Three scenarios for class-incremental learning.

	Freeze ConvNets	Freeze LSTM	Distillation type	Update first classifiers
3Stream-D	Yes	Yes	—	No
3Stream + cD	Yes	No	cD	Yes
3Stream + cD + tfD	Yes	No	cD + tfD	Yes

**Table 2:** Class-incremental action recognition performance on UCF and HMDB datasets.

Methods	Exemplar	UCF101			HMDB51	
		10 × 5 stages	5 × 10 stages	2 × 25 stages	5 × 5 stages	1 × 25 stages
UCIR [53]	5	74.31	70.42	63.22	44.90	37.04
PODNet [54]	5	73.26	71.58	70.28	44.32	38.76
TCD [7]	5	74.89	73.43	72.19	45.34	**40.07**
3Stream + cD	5	80.65	78.33	76.84	52.35	45.63
3tream + cD + tfD	5	**83.24**	**81.24**	**79.2**	**54.28**	47.15
3Stream-D	0	69.34	64.32	59.86	45.23	—
3Stream + cD	0	74.23	69.51	67.12	48.2	—
3tream + cD + tfD	0	*77.05*	*74.12*	72.07	*50.75*	—

**Table 3:** Class-incremental action recognition performance on Kinetics-400 dataset. Mean-named columns show the average recognition accuracy (%) of all incremental learning steps. The other values report the recognition accuracy of one model.

Exemplar	Base model	20 × 5 stages		10 × 10 stages		5 × 20 stages		Static mode
		Methods	Mean	Final	Mean	Final	Mean		Final
3Stream + cD	15	73.5	64.42	60.26	63.02	58.34	60.74	55.23	69.84
3Stream + cD + tfD	15	73.5	68.25	**65.78**	66.83	63.36	64.86	61.73	69.84
3Stream-D	0	73.5	55.3	46.32	52.26	43.54	48.73	39.6	69.84
3Stream + cD	0	73.5	60.4	56.86	58.7	54.21	55.38	51.63	69.84
3Stream + cD + tfD	0	73.5	63.9	**61.42**	62.43	60.12	60.9	58.35	69.84

**Table 4:** Effect of initialization on class-incremental action recognition accuracy on UCF101 dataset.

Exemplar	Methods	Random		Similar classifiers mean	
		10 × 5 stages	5 × 10 stages	10 × 5 stages	5 × 10 stages
3Stream + cD	5	70.14	67.23	71.32	68.86
3Stream + cD + tfD	5	75.31	72.7	76.47	73.76
3Stream-D	0	52.67	49.0	53.24	49.63
3Stream + cD	0	61.45	57.36	62.56	58.5
3Stream + cD + tfD	0	65.27	62.64	66.84	63.74

**Table 5:** Effect of spatial features distillation on class-incremental action recognition accuracy. Only the accuracy of the final model has been reported.

Methods	UCF101	Kinetics-400	HMDB51
	10 × 5 stages	20 × 5 stages	5 × 5 stages
3Stream + cD	62.56	56.86	36.43
3Stream + cD + tfD	66.84	61.42	40.26
3Stream + cD + tfD + sfD	**68.17**	**62.97**	**41.14**

## Data Availability

The datasets used to support the findings of this study are included in the article [48–50].
